# The effect of training on the perceived approach angle in visual vertical heading judgements in a virtual environment

**DOI:** 10.1007/s00221-020-05841-8

**Published:** 2020-06-08

**Authors:** Molly E. Gibson, John J.-J. Kim, Meaghan McManus, Laurence R. Harris

**Affiliations:** 1 Centre for Vision Research, York University, Toronto, ON Canada; 2 Department of Psychology, York University, 4700 Keele St, Toronto, ON M3J 1P3 Canada

**Keywords:** Vertical heading judgments, Training, Angle of approach, Flight simulator

## Abstract

Past studies have found poorer performance on vertical heading judgement accuracy compared to horizontal heading judgement accuracy. In everyday life, precise vertical heading judgements are used less often than horizontal heading judgements as we cannot usually control our vertical direction. However, pilots judging a landing approach need to consistently discriminate vertical heading angles to land safely. This study addresses the impact of training on participants’ ability to judge their touchdown point relative to a target in a virtual environment with a clearly defined ground plane and horizon. Thirty-one participants completed a touchdown point estimation task twice, using three angles of descent (3°, 6° and 9°). In between the two testing tasks, half of the participants completed a flight simulator landing training task which provided feedback on their vertical heading performance; while, the other half completed a two-dimensional puzzle game as a control. Overall, participants were more precise in their responses in the second testing compared to the first (from a SD of ± 0.91° to ± 0.67°), but only the experimental group showed improvement in accuracy (from a mean error of − 2.1° to − 0.6°). Our results suggest that with training, vertical heading judgments can be as accurate as horizontal heading judgments. This study is the first to show the effectiveness of training in vertical heading judgement in naïve individuals. The results are applicable in the field of aviation, informing possible strategies for pilot training.

## Introduction

Humans use optic flow to determine their direction of motion, also known as their heading (Gibson 1979). The majority of heading-focused optic flow research has investigated the ability to use visual motion to determine the direction of motion across a horizontal plane (see Vaina et al. 2004). Using optic flow, humans are able to discriminate their lateral heading within about 1°, which is sufficient for guiding walking or driving a car (Warren et al. 1988; Van den Berg 1992; Warren and Kurtz 1992). Precise vertical heading is normally less of a concern in everyday life as people generally cannot control their vertical heading. However, it is an important factor, especially in the sagittal plane, for pilots landing an aircraft. Optic flow cues such as the focus of expansion, the point from which the movement of all points in the field expand out during linear self-motion, are useful when landing an aircraft (Gibson et al. 1955) and for determining the timing of the ‘flare’, the point where a pilot ‘levels out’ to avoid impacting the ground (Palmisano et al. 2008). Studies show that humans are less accurate at judging vertical heading with discrimination thresholds of 2.5°–3° (Palmisano and Gillam 2005) and also less precise (Palmisano and Gillam 2005; MacNeilage et al. 2010) than horizontal heading. This lack of accuracy in vertical heading judgements is worrying since a vertical error of 3° on an approach path for an airplane would result in the pilot completely missing the runway. Pilots are required to land within a couple hundred feet of their intended landing position on a regular basis, which requires less than a 2.5° error (Transport Canada 2019). In usual circumstances, pilots have access to sophisticated instruments to assist in the landing process but if these fail, it may occasionally be necessary to rely on perceptual judgements. In the present study, we looked at the effect of training on vertical heading discrimination in sagittal plane using touchdown point estimation task.

Palmisano and Gillam (2005) explored vertical heading discrimination thresholds and the effects of different ground textures on the accuracy of approach angle judgment using a vertical heading detection threshold task. In their study, participants viewed a simulated descent on a computer screen. After the motion stopped, they had to indicate whether a red probe displayed on the simulated ground was above or below the touch down point. Participants were generally biased towards a five-degree approach, that is approaches steeper than 5° were judged as shallower and approaches shallower than 5° were judged as steeper than the real approach angle. Errors were decreased by adding visual cues such as an explicit horizon or randomly placed dots in addition to a runway outline (Palmisano and Gillam 2005).

Pilots are routinely able to land planes which suggests that they can determine vertical heading more accurately than the untrained population. So, how are pilots able to consistently land with a high level of precision despite inaccurate vertical heading estimates? Typically, pilots have access to instruments such as altitude and attitude indicators, which relay information from sensors, to help make corrections based on these instruments as well as visual information as they are descending. Additionally, pilots are required to go through hundreds of hours of training to be able to correctly and safely fly a plane. In the study by Palmisano and Gillam (2005), all participants received training on landing approaches through the Microsoft flight simulator landing tutorial prior to attempting the experimental task. However, no baseline performance was recorded prior to training, making it unclear whether their findings reflected natural human performance. Visual heading discrimination training has been shown to improve self-motion discrimination (Hartmann et al. 2013). Hartmann et al. (2013) trained participants to discriminate between linear leftward and rightward physical translation using a two-alternative forced choice task. Participants had to indicate in which direction they had moved after being translated laterally while sitting on a motion platform. Hartmann et al. (2013) recorded self-motion velocity thresholds of the participants before and after training phase. When training was done in the dark, blindfolded, participants’ performance did not improve despite extensive training (12 sessions with 40 min each), but performance did improve when visual input was provided during the training phase. The improvement was only found for the trained motion (linear leftward/rightward). Might vertical heading discrimination also be improved by visual training?

Processing of the vertical and horizontal components of self-motion appears to be segregated in the brain (Indovina et al. 2013) with dedicated systems for motion in each plane. There are more cells sensitive to visual motion in the lateral direction than there are for the vertical direction in the medial superior temporal (MSTd) area (Gu et al. 2010) and the otolith system, which detects the accelerations normally associated with self-motion, also has relatively greater sensitivity to horizontal motion compared to vertical (Rosenhall 1972). Indovina et al. (2013) had participants in an MRI scanner watch videos of simulated rollercoaster rides. They found that horizontal motion elicited more activation from medial temporal regions such as the para-hippocampus than was evoked by vertical motion. Given the non-uniform heading direction preferences in both visual and vestibular motions, it is plausible that human brain is less sensitive in general to self-motion in the vertical plane compared to the horizontal. It is possible that this relative lack of neural resources might set a lower limit on the effectiveness of training vertical heading accuracy compared to the improvements noted in horizontal heading,

Since previous research on landing approaches has focused on already-trained participants (Palmisano and Gillam 2005; Gibb et al. 2008; Kim et al. 2010), humans’ innate ability to gauge approach angles, i.e., vertical heading direction in the sagittal plane, and the effect of training on such judgements, is still unknown. During training, pilots are taught to use the focus of expansion as their ‘aim-point’ (Palmisano et al. 2008) and to use this point to gauge their rate of descent. Closely following Palmisano and Gillam’s (2005) vertical heading detection threshold task, we measured people’s baseline vertical heading judgement ability prior to providing training on a flight simulator program. We then remeasured their performance after the training. A second group of participants played a computer game of comparable cognitive difficulty instead of training as a control. If the ability to gauge an approach angle was to be improved by training, it would suggest that vertical heading judgements are typically less accurate than lateral ones because of lack of experience in this dimension. If vertical heading judgement ability was instead limited only by innate factors, then we would not expect training to improve vertical heading judgement accuracy.

## Methods

### Participants

38 participants (15 males, mean age = 20.1 years, SD = 2.3 years) were recruited from the York University Undergraduate Research Participant Pool (URPP) and were given course credit for participating in the study. All participants reported normal vision or used their prescribed vision correction for the duration of the study. Participants were also screened to ensure they had no previous flight training experience, either in an aircraft or in a simulator, and tested for stereoscopic acuity (using the Vision Assessment Corporation Fly Stereo Acuity Test with Lea Symbols P/N 1000) and color perception (using the abbreviated Ishihara color plate test). The study received ethics approval from the Glendon Psychology Delegated Research Ethics Review Committee prior to data collection and adhered to the Declaration of Helsinki. All participants signed a consent form prior to participating.

### Tasks

#### Main task

**Fig. 1:**
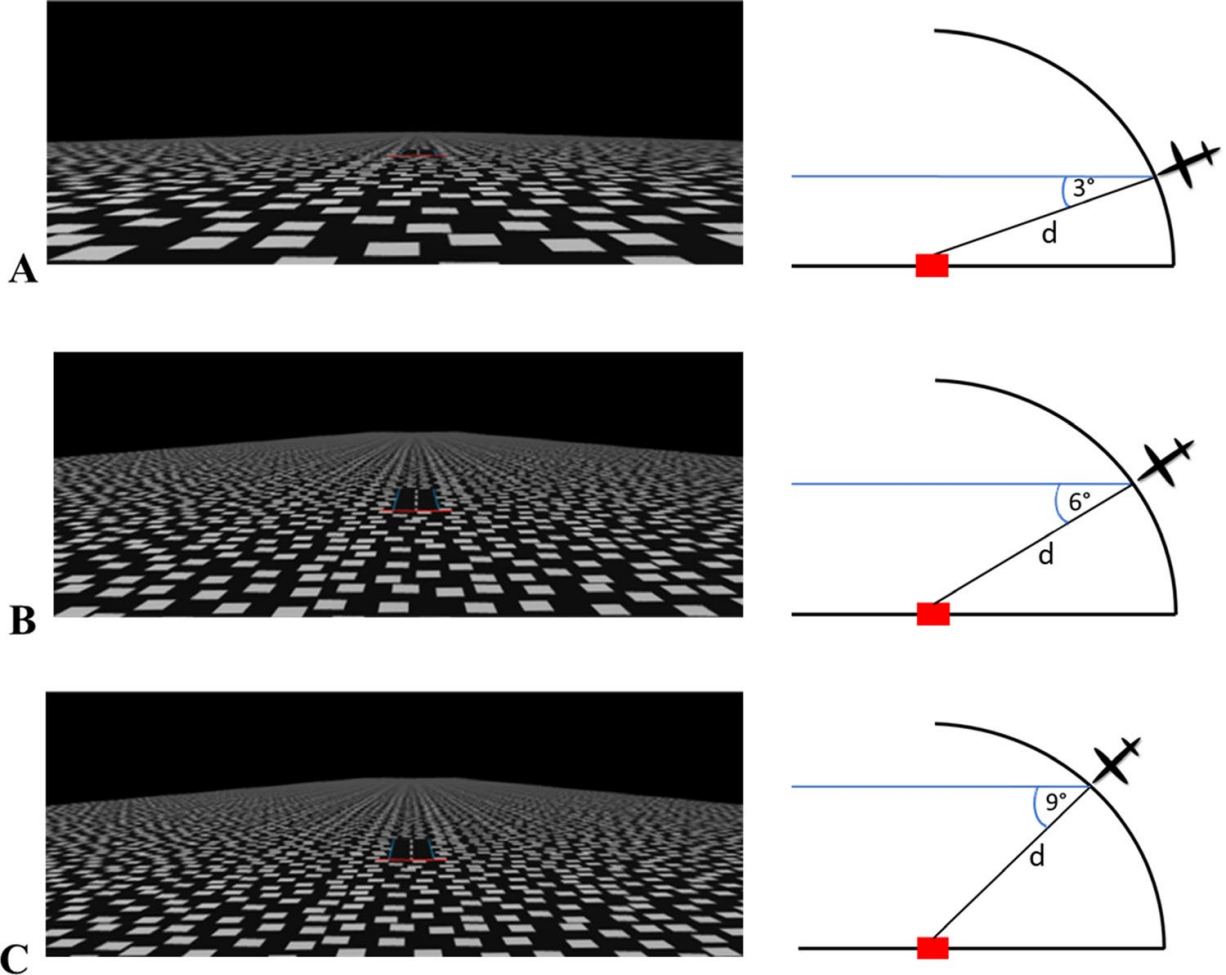
Participant’s view of **a** a three-degree approach angle, the height of the camera was 31.4 m, **b** a six-degree approach angle, the height of the camera was 62.7 m, and **c** a nine-degree approach angle, the height of the camera was 94.0 m, in the main task. The angles are measured from the camera view down, as shown in an exaggerated fashion in the insets on the right. The camera always started at a fixed distance from the target (*d* = 600 m). The figures on the right are not to scale

The main task was a touchdown point estimation task where a visually simulated aircraft landing descent was displayed binocularly in an Oculus Rift CV1 at a rate of approximately 90 frames per second. An adjustable-height chin rest was used to stabilize the participant’s head during the task. The task was programmed in Unity (version 5.3.8) and consisted of a series of two-second displays (180 frames) of simulated descents which displayed a ground plane of randomly placed, non-overlapping white squares on a black background continuing to infinity to create a horizon. A horizontal, red target line (*x* = 75 m, *y* = 1.5 m, *z* = 4.5 m) was drawn on the ground plane. Each trial started with a 1 s static view of the virtual environment from one of three start locations. The start locations corresponded to viewing angles of 3°, 6°, or 9°, referred as ‘target angles’, at 600 m away from the target (see Fig. 1 for detailed dimensions) where the viewer would land exactly on the red target line if they were to move straight. This static viewing was followed by a simulated descent at 75 m/s for 2 s. At the end of the two-second movement display, the screen went black and the participant was prompted by a static text display to respond as to whether they thought they would touch the ground before or after the red target line (press left mouse button for before, or right button for after). This was a forced choice where the participant had to pick one of the two answers. After the participant’s response the next trial began.

At each starting location, there were two initial angles of decent, one with a shallower angle of descent than the target angle (i.e., closer to 0° or horizontal) and the other with a steeper angle, bracketing each of the three target angles. The starting angles of decent were generated by adding or subtracting a randomly generated value between 1.5° and 3° to or from the target angle. For each of the six starting angles, the angle of descent was varied depending on the participant’s responses to the previous trial using a staircase function following a parameter estimation by sequential testing (PEST) method (Taylor and Creelman 1967). The PEST method honed in on the angle at which participants were equally likely to judge the vertical heading direction as too shallow or too steep to hit the target. The angle of descent was limited to a maximum of 20° and a minimum of  − 10°. If the participant’s response moved the angle of descent outside this range, then the same angle was displayed again. The six staircase functions (2 starting angles × 3 target angles) were randomly interleaved. The staircase functions were terminated at 20 trials resulting in a total of 120 trials per main task.

#### Experimental group—training task

**Fig. 2:**
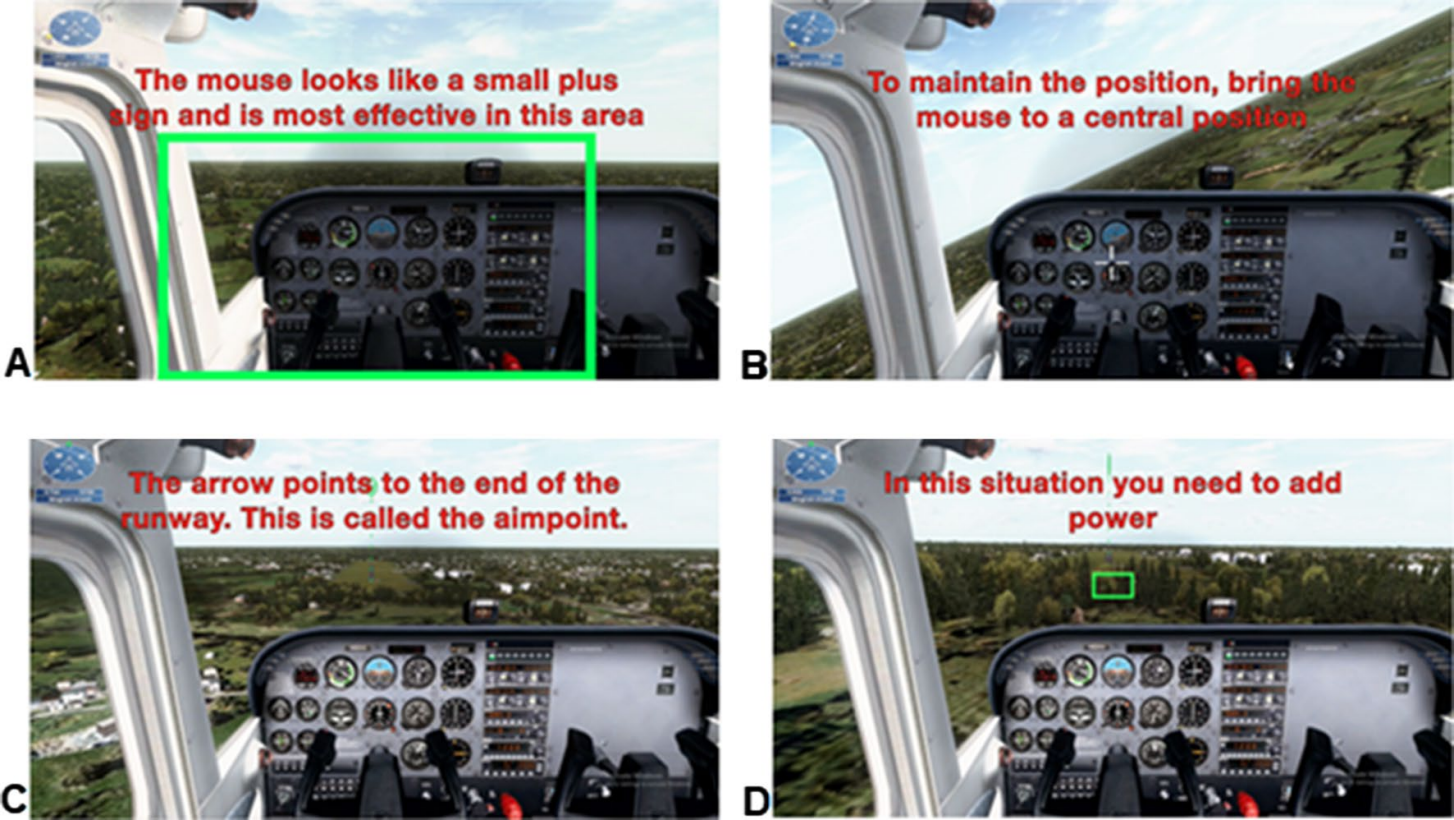
Screenshots from the instruction video displaying **a** an introduction to controlling the simulated aircraft, **b** the effects of the mouse, **c** introduction to the concept of the aim-point, and **d** the effects of power on the approach. The full video may be found at the following link: (https://youtu.be/yDye_P9sMB8)

The experimental group received training by watching an instruction video and then completing a series of landing missions in Microsoft Flight Simulator X: Steam Edition (FSX:SE) after first doing the main task. Screenshots from this video are shown in Fig. 2. Both the instruction video and the mission series were presented on a DELL U2414H screen at 60 frames per second with the participants seated approximately 45 cm from the screen. The instruction video was filmed using screen capture software and edited using iMovie (version#10). The landing missions were created using the FSX Mission Editor 2 (FSX extension software).

During the training phase, the experimental group viewed the instruction video which demonstrated the basic flight controls using the mouse and the up/down arrow keys as well as introducing the concept of an aim-point. The aim-point is the spot on the ground where the plane would impact the ground if a flare was not initiated. It is used to help pilots gauge their approaches (Wiener and Nagel 1988). Following the instruction video, the participant attempted a series of six approaches in Microsoft Flight Simulator which consisted of: two normal approaches (approximately a five-degree approach), two high approaches (a steeper-than-five-degree approach), and two low approaches (a shallower-than-five-degree approach). These approaches were presented in a random order with the exception that the normal approaches occurred first and last. To successfully complete the flight simulator training, the participant had to receive a success message following each of the six approaches. This message was displayed if the participant touched the ground within 200 feet of the aim-point in the simulator. If the participant failed to receive a success message, the failed mission was repeated until a success message was achieved.

Most participants completed the training in approximately 30 min. If the participant was unable to complete all six missions within 45 min, their training session was ended and their participation in the study was concluded. Two participants were unable to complete the training within the allotted time and were excluded from any statistical analyses. Of the remaining participants, it took them on average approximately two attempts to pass each approach (*M* = 2.05, SD = 0.97) with more attempts on the first trial (*M* = 3.89, SD = 2.35) compared to the 6th and final approach (*M* = 1.28, SD = 0.58).

#### Control group—cognitive task

**Fig. 3:**
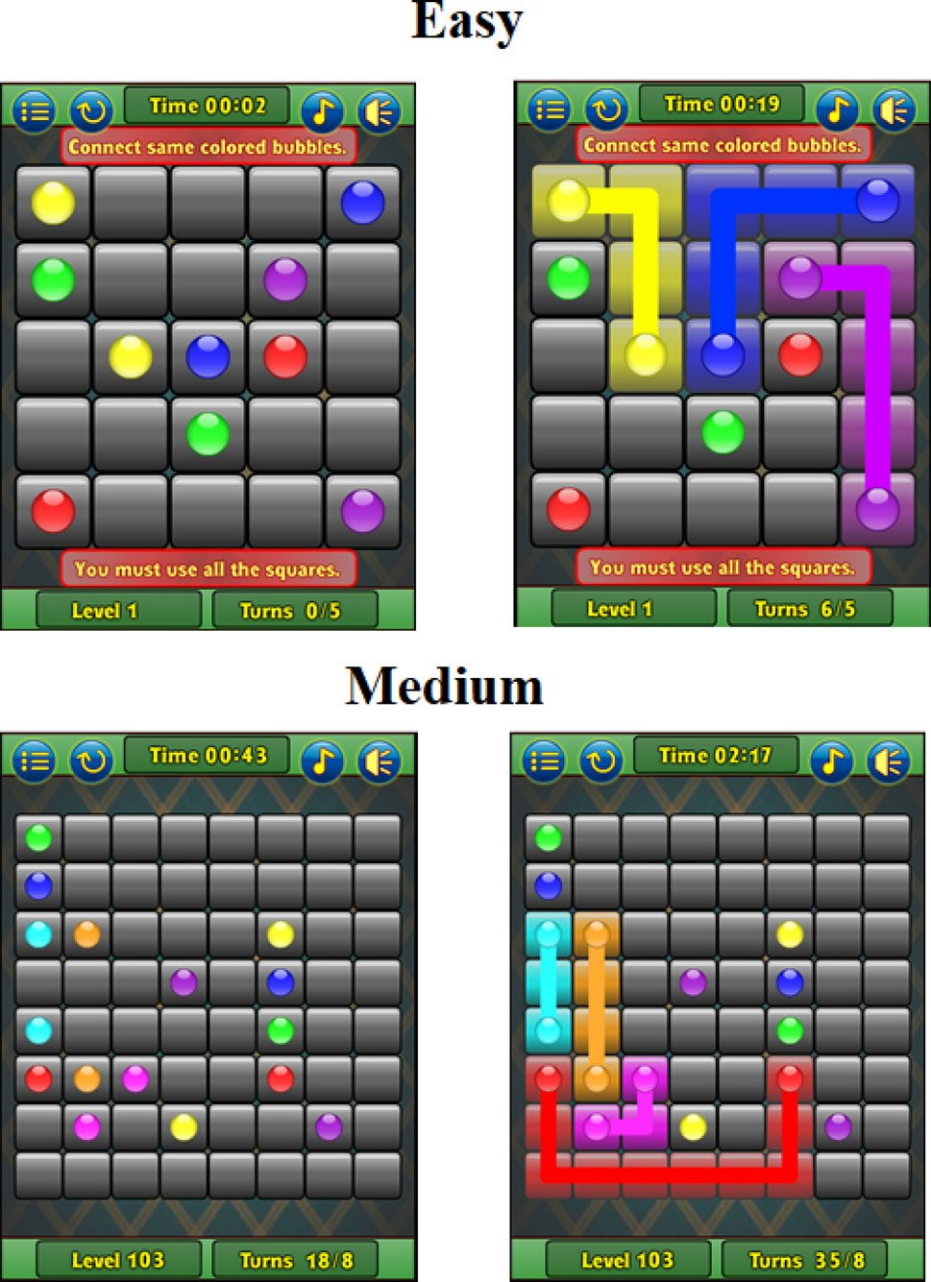
A demonstration of the control task game. The top row is the very first ‘easy’ level. The bottom row is a randomly selected ‘medium’ level (number 103)

The control group was assigned an alternate cognitive task which consisted of a web-based puzzle game presented on the same screen and using the same seating setup as the training task. The alternate task was a web-based game called Flow Free which involved connecting colors together in a grid in such a way that the lines did not cross and all the space in the grid was filled. For this task, the participants completed 10 levels of the easy level and then continued solving puzzles at the medium level until 30 min had passed (see Fig. 3). This game can be found at (http://playplayfun.com).

### Procedure

Participants were randomly assigned to either the experimental or control group. Each participant began the study by completing the screening process for normal vision using the materials outlined above. The participant was then comfortably fitted with the Oculus CV1 and chinrest and then performed the main task which took approximately 10 min. Following this, the participant entered the training phase. If assigned to the experimental group, the participant completed the training task. If assigned to the control group, the participant completed the cognitive task. Both tasks took approximately 30 min to complete. Once the training or cognitive task was complete, each participant then completed the main task a second time.

### Data analysis

**Fig. 4:**
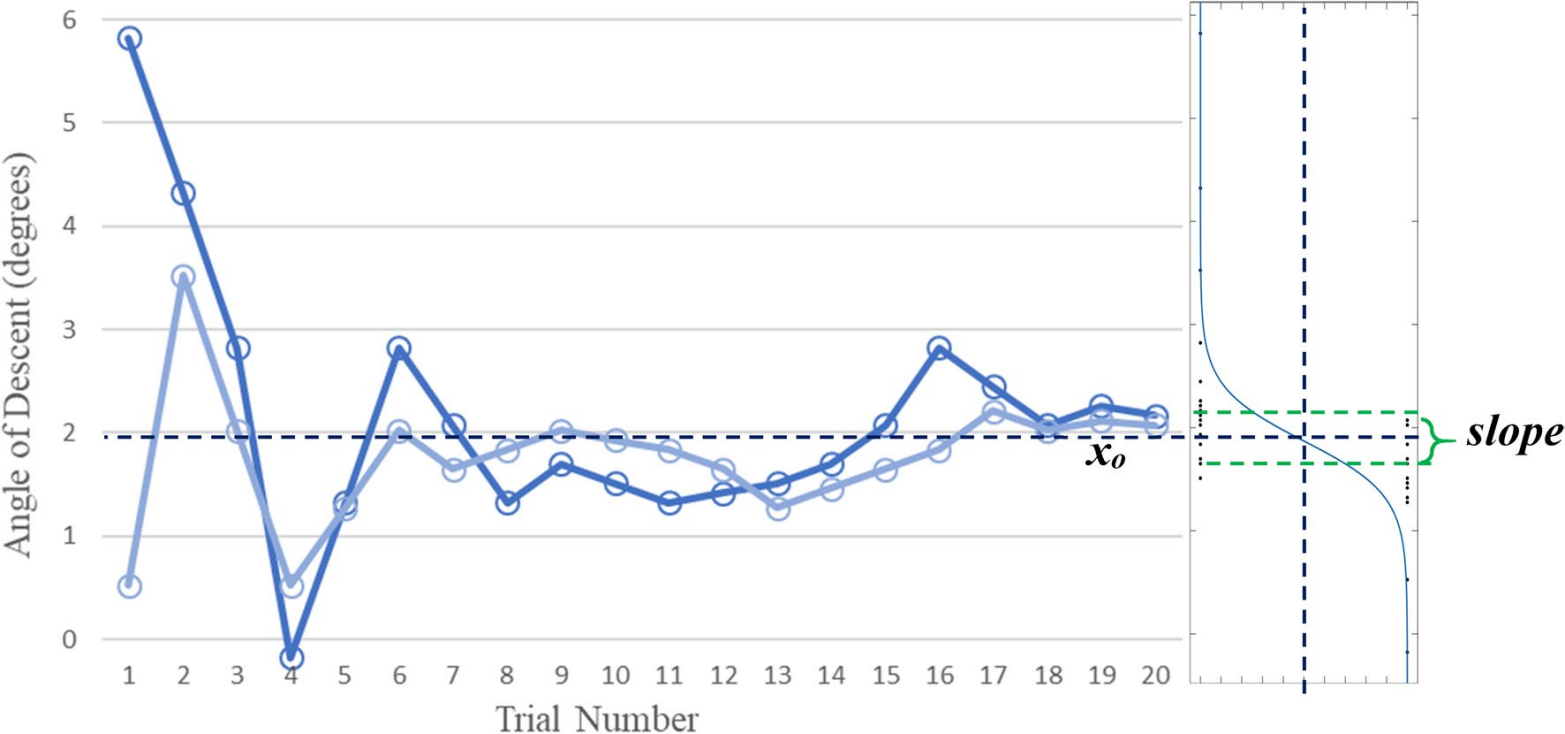
Sample of data collected from staircases for a single target angle from a typical subject (responses for target angle of 3°) using the PEST method (left) as a function of trial number, and the best-fit logistic curve fitted to those data using Eq. 1 (right): the dashed horizontal line corresponds to *x*_0_ = the visual vertical heading angle judged as equal to the target angle, 1/*b* = estimate of the slope of the curve. The individual responses for each angle of descent are shown as small dots in the right-hand part of the figure

A typical example of the data collected is shown in Fig. 4. Best-fit logistic functions were fit to the combined staircase data for each angle for each participant (Eq. 1) (using 0 for overshoot and 1 for undershoot).

1 $${\text{Response}} = \frac{1}{{1 + {\text{e}}^{{\left( { - \frac{{\left( {x - x_{0} } \right)}}{b}} \right)}} }},$$

where *x* is the actual angle of descent, *x*_0_ is the angle judged as equal to the target angle (accuracy) and *b* is inversely proportional to precision.

We analyzed the vertical heading angles (accuracy) and the precision using a mixed-design ANOVA using IBM SPSS. The analysis consisted of the relation between groups (experimental and control), test sessions (pre- and post-training phase), and target angles (3°, 6° and 9°).

#### Outliers

Seven participants out of the 38 total participants (19 in both groups) were identified as outliers and excluded from the statistical analyses, leaving 31 participants (16 in the experimental group and 15 in the control group). Our methods for identify outliers were as follows:

First, we looked at the participants’ performance in their staircase functions. Four participants reached the angle limits of the training program (+ 20° or − 10°) on one or more of their staircase functions. One participant hit the limit around trial 7 and then appeared to have tried to correct for it but never recovered. These participants (two from the experimental group and three from the control group) seem to have misunderstood the instruction and used wrong buttons for their responses during the task. Their data were removed and not used in the analysis.

Second, we evaluated the distribution of the vertical heading angles of the remaining participants. If participant’s average heading angle fell in the extreme tail ends of the distribution, where it differed from 99% of the rest of the data, the person was considered as an outlier. As a result, the data from one person from the experimental group were removed.

Lastly, one additional participant was removed as they were unable to distinguish heading angles and appeared not to understand the task. The two staircase functions used for the same target angle did not converge, resulting in more than a 5° difference between the angles of descent in the final trial of the two functions (trial# 20, see Fig. 4 as a reference). The data from this person, from the control group, were removed from the analysis.

## Results

### Accuracy

**Fig. 5:**
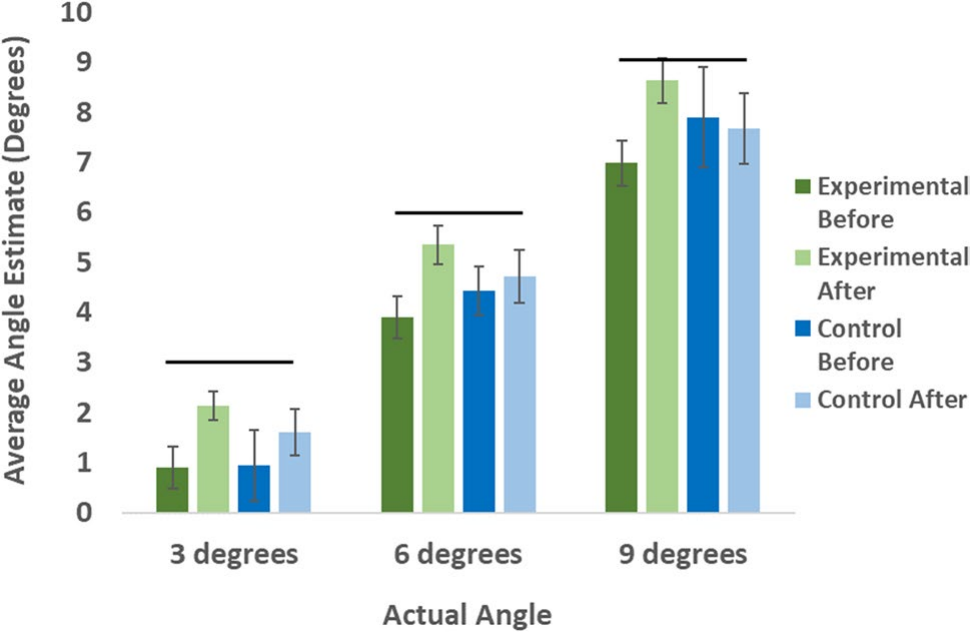
The mean vertical heading angles for the experimental (green) and control (blue) groups before (dark) and after (light) either the training (experimental group) or control task (control group) for each approach angle. Solid horizontal bars indicate the correct response. Error bars are ± 1 standard error

There was a main effect of target angle, *F* (1.22, 35.25) = 315.08, *p* < 0.001, *η*_*p*_^2^ = 0.916. A post hoc analysis using Bonferroni correction found that the mean heading angles for each target significantly differed from one another (*p* < 0.001 in all cases): 1.39° (SE = 0.32°) for the 3° target, 4.60° (SE = 0.30°) for the 6° target and 7.80° (SE = 0.46°) for the 9° target (see Fig. 5).

A main effect of test session was found, *F* (1, 29) = 16.70, *p* < 0.001, *η*_*p*_^2^ = 0.365. Participants’ mean heading angles before and after the training phase were 4.17° (SE = 0.39°) before and 5.02° (SE = 0.31°) after. For this average score, a mean of 6° would have been a perfect performance.

The interaction between test sessions and groups was significant, *F* (1,29) = 8.58, *p* = 0.007, *η*_*p*_^2^ = 0.228. We followed up with a post hoc test with Bonferroni correction which revealed that the mean heading angle was significantly higher after the training phase compared to before for the experimental group (*p* < 0.001) but not for the control group (*p* = 0.427). There was no significant interaction found between target angles and groups, *F* (1.22, 35.35) = 0.113, *p* = 0.788, *η*_*p*_^2^ = 0.004, or between test sessions and target angles, *F* (1.64, 47.69) = 0.372, *p* = 0.650, *η*_*p*_^2^ = 0.013 (see Fig. 5). Lastly, the three-way interaction between test sessions, target angles, and groups was not significant, *F* (1.64, 47.68) = 2.99, *p* = 0.070, *η*_*p*_^2^ = 0.093. To further evaluate the constant underestimation found for the required approach angle to reach each target, we conducted post hoc t-tests for each condition (a total of four tests). The results are shown in Table 1 below.

**Table 1:** The results of four one sample *t* tests (two tailed), where the errors participants made for each condition were compared to 0 (perfect)

Condition	Overall mean error (SD)	*T*	*N*	*p* value
Control (pre-training)	− 1.6 (2.9)	− 3.615	45	0.001
Experimental (pre-training)	− 2.1 (1.7)	− 8.538	48	< 0.001
Control (post-training)	− 1.3 (2.1)	− 4.144	45	< 0.001
Experimental (post-training)	− 0.6 (1.5)	− 2.890	48	0.006

The error participants made for each condition was computed by subtracting the target angle from the heading angle. All errors for target angles of 3°, 6° and 9° were then collapsed by averaging the errors for each target angle to get the mean errors for each participant for each condition. The Alpha level required for significance after correcting for the multiple comparisons (Bonferroni correction) is *p* < 0.012

### Precision

**Fig. 6:**
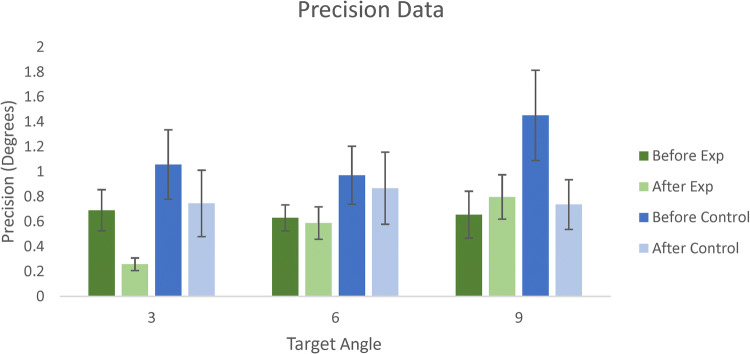
The mean precision (slope of the logistic fits) for each angle for the experimental (green) and control (blue) groups before (dark) and after (light) either the training (experimental group) or control task (control group) for each approach angle. 0° on the *Y* axis represents perfect performance. Error bars are ± 1 standard error

Precision was estimated by deriving the slope of the curve fit to the staircase data for each angle for each participant using the logistic function (1/*b* from Eq. 1; see Fig. 4). There was a main effect of test session, *F* (1, 29) = 6.504, *p* = 0.016, *η*_*p*_^2^ = 0.183, where the slopes of the logistic for each participant’s decisions during the main task before and after the training phase were 0.91° (SE = 0.12°) and 0.67° (SE = 0.11°), respectively. All other main effects and interactions were not significant (see Fig. 6).

## Discussion

To know where they would land when experiencing a decent either simulated or real, a person needs to know their angle of decent, i.e., their vertical heading angle. This study was the first to test untrained humans on a touchdown point estimation task in the sagittal plane and to then explore the effectiveness of visual training on these judgements of where they would touchdown. The accuracy of the experimental group, but not the control group, improved significantly after training. The experimental group became accurate to within 1° after training (see Fig. 5; Table 1), but there was no statistically significant change in accuracy for the control group. This finding suggests that training is important for accurate vertical heading estimation. The non-significant interaction between target angle and group, or target angle and test session also show that the errors made were not influenced by target angles despite the fact that the initial heights and horizontal distances for these target angles differed (see Fig. 1). Interestingly, the errors in vertical heading estimates still significantly differed from 0 (*M* = − 0.6°, SD = 1.5°, see Table 1) indicating a constant underestimation of the heading angle needed to land on the target. Participants consistently chose an angle of descent that was too shallow, leading to overshooting the target. These findings are in line with Palmisano and Gillam (2005) who also trained participants before a vertical heading detection threshold task but did not report pre-training performance levels. In our current experiment, participants were assessed before and after training for less than an hour using a visual simulation on a monitor.

Training did not improve performance for any specific target angle more than any other. Instead, the improvement for each target angle (3°, 6°, and 9°) was relatively consistent, resulting in a similar error regardless of the target angle (see Fig. 5). This is in contrast to the study by Palmisano and Gillam (2005) who found that their participants were most accurate at approaches close to 5°. This is likely due to the fact that our study used a different training procedure to that employed in their study. In the current study participants received training on a variety of angles in the simulated landing approach task (or no training at all) while in Palmisano and Gillam’s study, all participants received training on the standard approach in Microsoft Flight Simulator. This means that Palmisano and Gillam’s study may only have increased participants’ accuracy for approach angles around the angle used by the Microsoft Flight Simulator (2002 version): the only angle on which they were trained. However, since no baseline is reported in the Palmisano and Gillam (2005) paper, we cannot be sure.

Evaluation of participants’ precision revealed that the variability of their responses reduced the second time that they did the main task, presumably as the result of increased familiarity with the task (less noisy responses). However, no specific effect of training on their precision was evident as similar improvement was found in both groups.

**Fig. 7:**
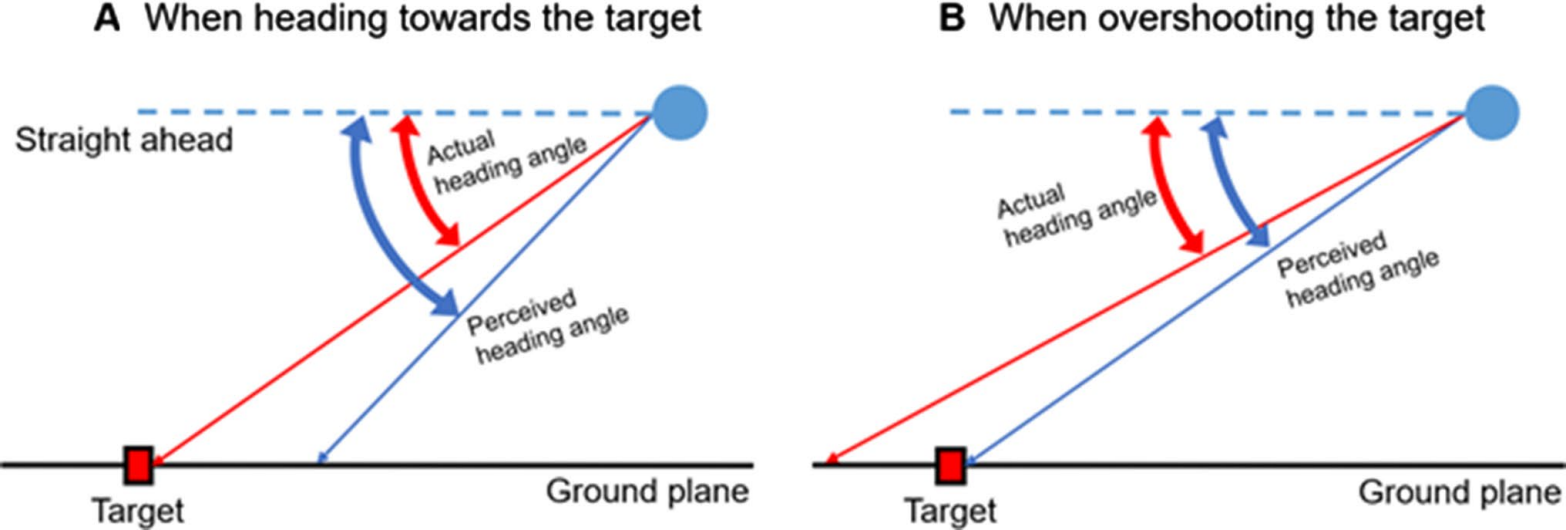
Participant’s perceived heading angle is biased away from the straight ahead. When they are descending along the actual, or correct angle towards the target they perceive themselves as undershooting the target (**1**). When they are overshooting the target, descending at too shallow of an angle, they perceive themselves as hitting the target (**2**). In this task, overshooting the target might reflect a bias in the perceived direction of travel away from the straight ahead

Differences in the numbers of cells in the medial temporal region and elsewhere processing vertical heading (Indovina et al. 2013) may underlie lower precision in vertical compared to horizontal heading judgements. However, the bias in accuracy we find, namely the tendency to overshoot the target in untrained humans, may reflect a general bias to displace the perceived direction of travel away from the straight ahead as illustrated in Fig. 7, where participants need to overshoot the target to feel they will hit it. Crane (2014) tested vertical heading estimation every 5° for all 360° in the sagittal plane and also found direction-specific biases away from the straight ahead. His participants were fairly accurate in visual heading estimation over the range of angles of descent we used (93°–99° in his convention), although there was a trend towards errors away from straight ahead (see Fig. 7c, p. 95; Crane 2014). Such a bias away from straight ahead has also been reported for horizontal heading estimations (Crane 2012; Cuturi and MacNeilage 2013; Hummel et al. 2016; Winkel et al. 2018). Such a bias might arise due to the anisotropy in MSTd (Gu et al. 2010) resulting in neurons responding more strongly for headings that deviate from straight ahead.

Humans do not normally receive feedback about errors in their vertical heading judgements compared to the obvious and immediate consequences of misjudgments of horizontal heading. Vertical heading judgements after training had an average error of only 0.6° which is similar to that reported for horizontal visual heading tasks (Warren et al. 1988; Warren and Kurtz 1992). The fact that only 45 min of training can bring performance into line with the performance of previously reported horizontal heading tasks is encouraging. Perhaps longer training sessions, possibly including multimodal sensory inputs, could result in participants becoming even more accurate. The improvement we found indicates that humans may be able to detect errors in vertical heading just as well as errors in lateral heading when given adequate training and exposure, at least over the range of vertical heading angles we tested. Since our participants performed the touchdown point estimation task immediately after training, we have no idea how long their improved accuracy might have lasted. Further study is needed to evaluate whether this improvement in judging visual vertical heading could be enhanced further, how long it lasts, and whether it can be transferred to the ability to land an aircraft more accurately.
